# CRISPR-Cas Dynamics in Carbapenem-Resistant and Carbapenem-Susceptible *Klebsiella pneumoniae* Clinical Isolates from a Croatian Tertiary Hospital

**DOI:** 10.3390/pathogens14060604

**Published:** 2025-06-19

**Authors:** Ivana Jurić, Marko Jelić, Manda Markanović, Lucija Kanižaj, Zrinka Bošnjak, Ana Budimir, Tomislav Kuliš, Arjana Tambić-Andrašević, Ivana Ivančić-Baće, Ivana Mareković

**Affiliations:** 1 Clinical Microbiology, Infection Prevention and Control Department, University Hospital Centre Zagreb, Kišpatićeva 12, 10000 Zagreb, Croatia; manda.markanovic@kbc-zagreb.hr (M.M.); lucija.kanizaj@kbc-zagreb.hr (L.K.); zrinka.bosnjak@kbc-zagreb.hr (Z.B.); ana.budimir@kbc-zagreb.hr (A.B.); ivana.marekovic@kbc-zagreb.hr (I.M.); 2 Department of Clinical Microbiology, University Hospital for Infectious Diseases “Fran Mihaljević”, Mirogojska 8, 10000 Zagreb, Croatia; mjelic@bfm.hr (M.J.); arjana.tambic@bfm.hr (A.T.-A.); 3 School of Medicine, University of Zagreb, Šalata 3, 10000 Zagreb, Croatia; 4 Department of Urology, University Hospital Centre Zagreb, Kišpatićeva 12, 10000 Zagreb, Croatia; tomislav.kulis@kbc-zagreb.hr; 5 Molecular Biology Department, Faculty of Science, University of Zagreb, Horvatovac 102a, 10000 Zagreb, Croatia; ivana.ivancic.bace@biol.pmf.hr

**Keywords:** *Klebsiella pneumoniae*, CRISPR-Cas system, CRISPR loci, subtypes I-E and I-E*, carbapenemase genes, carbapenem resistance, antimicrobial resistance, PFGE, MLST

## Abstract

(1) Background: CRISPR-Cas systems provide adaptive immunity against mobile genetic elements (MGEs) carrying antimicrobial resistance (AMR) genes. Carbapenem-resistant (CR) *Klebsiella pneumoniae* is a major public health concern, and the role of CRISPR-Cas in its resistance is understudied. This study explored CRISPR-Cas associations with multidrug resistance in clinical *K. pneumoniae*. (2) Methods: 400 *K. pneumoniae* isolates (200 CR and 200 carbapenem susceptible (CS)) were analyzed. Carbapenemase genes (*bla*_OXA-48_, *bla*_NDM-1_, *bla*_KPC-2_), *cas1*, *rpoB*, and CRISPR1-3 loci were identified by PCR, while only CRISPR loci were sequenced. Genetic relatedness was assessed via PFGE, MLST, and spacer analysis. Statistical analysis utilized chi-squared and Fisher’s exact tests. (3) Results: CRISPR-Cas was present in 15.8% of isolates, mainly subtypes I-E and I-E* (93.3%), with CRISPR3 loci showing the greatest spacer diversity. Clonal complexes ST14/15/101 (CR) and ST35 (CS) were identified. *bla*_OXA-48_ was linked to CRISPR-Cas-negative strains, while *bla*_NDM-1_ and *bla*_KPC-2_ were more frequent in CRISPR-Cas-positive strains (*p* < 0.0001). Imipenem/relebactam resistance was higher in CRISPR-Cas-negative isolates. (4) Conclusions: *K. pneumoniae* CRISPR-Cas systems correlate with specific carbapenemase profiles, suggesting pressure against *bla*_OXA-48_ acquisition. The coexistence of I-E and I-E* subtypes highlight synergies in targeting MGEs. CRISPR loci could be tools for subtyping organisms following MLST.

## 1. Introduction

*Klebsiella pneumoniae*, a Gram-negative species classified within the order Enterobacterales, is a significant opportunistic pathogen causing ~30% of hospital-acquired Gram-negative infections, including pneumonia, meningitis, bloodstream infections, and urinary tract infections [1,2]. The rise of extended-spectrum β-lactamase (ESBL)-producing Enterobacterales has driven carbapenem overuse, leading to carbapenemase-producing strains, particularly in hospital settings [1,3,4]. Carbapenem-resistant Enterobacterales (CRE), defined by resistance to ≥1 carbapenem, is clinically critical due to treatment delays and increased mortality [5,6]. Globally, 85% of CRE exhibit carbapenemase production as the primary resistance mechanism [7]. In Croatia, the resistance of *K. pneumoniae* to carbapenems is predominantly attributed to the presence of the *bla*_OXA-48_, *bla*_NDM-1_, *bla*_KPC-2_, and *bla*_VIM-2_ genes, with carbapenemase OXA-48 being the most prevalent among clinical isolates [8,9].

Carbapenemase-coding genes are often spread by horizontal gene transfer (HGT) on mobile genetic elements, mostly plasmids and transposons [10]. Despite the potential advantages of HGT, bacteria must balance acquiring beneficial traits with avoiding detrimental genetic elements that could deplete their energy resources. To limit the entry of invasive genetic elements, such as bacteriophages and plasmids, bacteria use clustered regularly interspaced short palindromic repeat and their associated *cas* genes (CRISPR-Cas) that provide bacteria adaptive immunity by preventing the acquisition of foreign DNA or RNA [11]. The CRISPR-Cas system has been detected in about 45.0% of bacterial genomes and is classified into two classes, six types (I–VI) and 33 subtypes [12,13]. A functional CRISPR-Cas system consists of a CRISPR locus, a cluster of associated *cas* genes, and a leader sequence. CRISPR loci typically consist of highly conserved repetitive sequences (21–48 bp) separated by unique spacers (21–72 bp). The leader sequence is located upstream of the first spacer and it acts as a promoter for the transcription of the CRISPR locus [14,15]. Three CRISPR-Cas system subtypes (I-E, I-F, and I-E*) have been detected in published *Klebsiella* genomes. According to their relative position on the chromosome, the CRISPR loci are labelled from CRISPR1 to CRISPR5. Subtype I-E, which contains the CRISPR-1 locus, and subtype I-E*, which contains the CRISPR2 and/or CRISPR3 loci, were found in *K. pneumoniae* [14].

Several studies have shown that *K. pneumoniae* isolates without a CRISPR-Cas system are more likely to acquire resistance genes, including carbapenemase, ESBLs, and genes coding for aminoglycoside-modifying enzymes (AME) [15,16,17,18]. Recent studies on clinical *K. pneumoniae* isolates revealed that these systems are not uniformly distributed among isolates but are explicitly associated with specific sequence types (STs), suggesting that it may play a role in the adaptation and virulence of particular *K. pneumoniae* strains [17,19,20]. Despite this, the link between CRISPR-Cas systems and antimicrobial resistance in *K. pneumoniae* remains unclear.

In this study, we aimed to investigate CRISPR-Cas prevalence in *K. pneumoniae* clinical isolates from the UHC Zagreb and examined its associations with department and sample distribution, patient clinical characteristics, antimicrobial resistance profiles, carbapenemase gene types, PFGE, and ST groups.

## 2. Materials and Methods

### 2.1. Ethics

This study received ethical approval from the University Hospital Centre Zagreb (UHCZ) Ethics Committee (approval class: 8.1-23/35-2; number: 02/013 AG) on 06 February 2023. All patient information was de-identified, and patient consent was not required. Patient data will not be shared with third parties.

### 2.2. Bacterial Isolates and Antimicrobial Susceptibility Testing

In this retrospective study, conducted from January 2021 to April 2024, 400 non-consecutive, non-duplicate clinical bacterial isolates of *K. pneumoniae* were collected from different hospital departments, as detailed in Table 1. UHCZ is a tertiary care hospital with 1148 beds and 56,723 admissions per year. It has 30 different clinical departments and 1,473,340 polyclinic patients per year. We identified the isolates using a “Bruker MALDI Biotyper” mass spectrometer (Bruker Daltonik GmbH, Bremen, Germany). The identified isolates were stored at −80 °C until used. Antimicrobial susceptibility testing was performed using the disk diffusion method according to the European Committee on Antimicrobial Susceptibility Testing (EUCAST) guidelines. Carbapenem resistance was defined as resistance to ertapenem (ETP). The presence of carbapenemases was initially detected by RESIST-5 O.K.N.V.I. immunochromatographic assay (Coris BioConcept, Gembloux, Belgium). In ETP-resistant isolates, minimum inhibitory concentration (MIC) was determined for imipenem (IPM), meropenem (MEM), and colistin (COL) by broth microdilution method, according to EUCAST guidelines [21].

### 2.3. Molecular Screening for Carbapenemase Genes and the CRISPR-Cas System

The DNA was extracted from cells lysed by boiling in water. Carbapenemase-encoding genes were identified using PCR amplification followed by 1.5% agarose gel electrophoresis as previously described [22,23]. Additionally, housekeeping gene *rpoB*, *cas1*, and CRISPR loci 1, 2, and 3 were detected with primers listed in Table 2 [17,24,25]. PCR products were purified by GeneJET PCR Purification Kit (Thermo Fisher Scientific, Waltham, MA, USA) and subjected to DNA sequencing (Macrogen Europe BV, Amsterdam, The Netherlands). The sequences of CRISPR arrays were identified manually and with CRISPRCasFinder, a bioinformatics web server for detecting CRISPR arrays and Cas proteins. This algorithm identifies direct repeat sequences ranging from 23 to 55 bp, which are interspersed with variable sequences of 25 to 60 bp. CRISPR arrays are categorized according to an evidence level, rated from 1 to 4, with level 1 encompassing small CRISPRs containing three or fewer spacers and levels 2 to 4 based on the similarity of repeats and spacers. Our analysis focused exclusively on those classified by the program as evidence levels 2 to 4, which are considered confirmed CRISPR loci. Subsequently, we examined each spacer for its identity in GenBank through a nucleotide BLASTn search.

### 2.4. Pulsed-Field Gel Electrophoresis and Multilocus Sequence Typing

Only CRISPR-Cas positive isolates were further investigated by XbaI-digested genomic DNA pulsed-field gel electrophoresis (PFGE) using the CHEF DR–III^®^ system (Bio-Rad Laboratories, Hercules, CA, USA) according to the standardized PulseNet protocol program, as described previously [26,27,28]. Representative isolates of PFGE groups were subjected to multilocus sequence typing (MLST) using seven conserved housekeeping genes (*gapA*, *infB*, *mdh*, *pgi*, *phoE*, *rpoB*, and *tonB*) according to the protocol described on the Institute Pasteur website [28,29]. A total of 39 isolates were typed with MLST. EBURST v3 was used to define ST groups and clonal complexes of analyzed clonal representatives of CRISPR-Cas-positive *K. pneumoniae* isolates, emphasizing the clonal spread of bacterial isolates [30].

### 2.5. Statistical Analysis

All *K. pneumoniae* isolates were classified as CRISPR-Cas positive or negative and compared across hospital departments, carbapenemase gene profiles, antimicrobial susceptibility patterns, PFGE, and ST groups. Statistical analysis was performed using chi-squared and Fisher’s exact tests, with a significance threshold of *p* < 0.05. Logistic regression was applied to assess the distribution of CRISPR arrays, and the Mann–Whitney test was used to compare patient age between groups. All analyses were conducted using MedCalc Statistical Software, v 20.0.4.

## 3. Results

### 3.1. Antimicrobial Susceptibility of Bacterial Isolates

**Figure 1:**
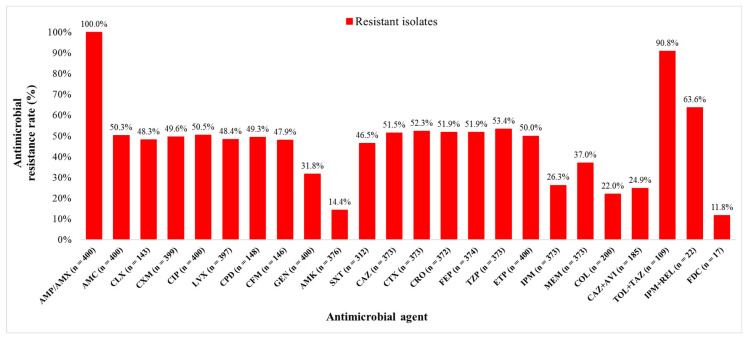
*Klebsiella pneumoniae* isolates resistance rates to different antimicrobial agents. AMP—ampicillin, AMX—amoxicillin, AMC—amoxicillin + clavulanic acid, CLX—cephalexin, CXM—cefuroxime, CIP—ciprofloxacin, LVX—levofloxacin, CPD—cefpodoxime, CFM—cefixime, GEN—gentamicin, AMK—amikacin, SXT—trimethoprim + sulfamethoxazole, CAZ—ceftazidime, CTX—cefotaxime, CRO—ceftriaxone, FEP—cefepime, TZP—piperacillin + tazobactam, ETP—ertapenem, IPM—imipenem, MEM—meropenem, COL—colistin, CAZ + AVI—ceftazidime + avibactam, TOL + TAZ—ceftolozane + tazobactam, IPM + REL—imipenem + relebactam, and FDC—cefiderocol.

Out of 400 *K. pneumoniae* isolates, 200 (50.0%) isolates were carbapenem resistant (CR) and 200 (50.0%) carbapenem susceptible (CS). The isolates originated from diverse clinical samples, most commonly urine (38.3%), rectal swabs (17.8%), and stool (9.3%). The median age of patients with CR and CS *K. pneumoniae* isolates were 66.5 (0–92) and 50.5 (0–92) years, respectively (*p* < 0.0001). Among all 400 patients, hypertension was the most common comorbidity (47.8%), followed by ICU stay (41.5%), malignant diseases (28.3%), and diabetes (21.0%). All CR *K. pneumoniae* isolates included in this study met the criteria for multidrug resistance (MDR). The bacterial isolates were tested for susceptibility to 24 different antimicrobial agents. Resistance rates to ETP, MEM, and IPM were 50.0% (200/400), 37.0% (138/373), and 26.3% (98/373), respectively. The lowest resistance rates overall were observed for FDC (2/17, 11.8%), amikacin (AMK) (54/376, 14.4%), and COL (44/200, 22.0%). Resistance rates of CR *K. pneumoniae* isolates to the new β-lactam/β-lactamase inhibitors were 90.8% (99/109) to TOL + TAZ, 63.6% (14/22) to IPM + REL, and 24.9% (46/185) to CAZ + AVI (Figure 1).

### 3.2. Distribution of Carbapenemase-Encoding Genes and the CRISPR-Cas System

Among five carbapenemase-encoding genes in CR *K. pneumoniae* isolates, the *bla*_OXA-48_ gene was the most common and was detected in 123 isolates (61.5%). The *bla*_NDM-1_ gene was detected in 53 (26.5%), *bla*_NDM-1_ and *bla*_OXA-48_ simultaneously in 14 (7.0%), and *_bla_*_KPC-2_ in 10 (5.0%) CR isolates. All KPC-producing isolates (10/10) were from 2021, the majority of NDM-1 producers (31/53, 58.5%) from 2022, while OXA-48 producers (alone or with NDM-1) were most common in 2023 and 2024 (91/137, 73.0%).

**Figure 2:**
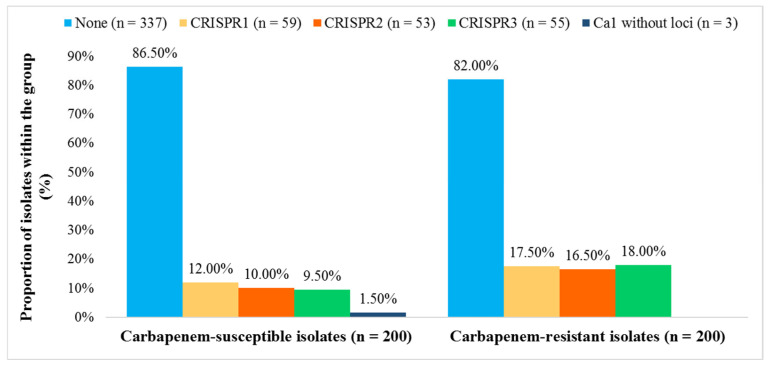
Distribution of CRISPR 1, 2, and 3 loci in carbapenem-susceptible and carbapenem-resistant *Klebsiella pneumoniae* isolates (*n* = 400).

We detected the *cas1* gene in 63 (15.8%) and CRISPR arrays in 60 (15.0%) isolates. Among these, 56 contained CRISPR1, CRISPR2, and/or CRISPR3 arrays (subtypes I-E and I-E*), three isolates exhibited only CRISPR1 (subtype I-E), while a single isolate was found to possess CRISPR3 (subtype I-E*). Three isolates that were positive for the *cas1* gene but lacked a detectable CRISPR array were also classified as CRISPR-Cas positive. CRISPR3 locus was significantly more frequent in CR *K. pneumoniae* isolates (OR = 2.03, 95.0% CI [1.154, 3.790]; *p* = 0.0150), whereas CRISPR1 and CRISPR2 did not differ significantly between CR and CS groups (CRISPR1: OR = 1.556, 95.0% CI [0.888, 2.727], *p* = 0.1228; CRISPR2: OR = 1.778, 95.0% CI [0.982, 3.221], *p* = 0.0575) (Figure 2).

CRISPR-Cas-positive isolates were significantly more frequent among patients admitted to the COVID-19 ICU (7/63, 11.1%) compared to CRISPR-Cas-negative isolates (5/337, 1.5%; *p* = 0.0007). Conversely, CRISPR-Cas-negative isolates were more prevalent in patients hospitalized in the pediatric ICU (50/337; 14.8%) compared to those with CRISPR-Cas-positive isolates (1/63, 1.6%; *p* = 0.0016). Furthermore, patients whose isolates harbored a CRISPR-Cas system had a significantly higher incidence of hypertension (65.1% vs. 44.5%; *p* = 0.0027) and diabetes (31.7% vs. 19.0%; *p* = 0.0227) compared to patients with CRISPR-Cas-negative isolates. No significant association was found between CRISPR-Cas presence and different clinical sample types, except for CRISPR-Cas-negative isolates being more frequent in stool samples (10.7% vs. 1.6%; *p* = 0.0172).

### 3.3. Correlation Between Antimicrobial Resistance and the CRISPR-Cas System

The frequency of the *bla*_OXA-48_ was significantly higher in the CRISPR-Cas-negative isolates (120/337, 35.6%; *p* < 0.0001). In contrast, *bla*_NDM-1_ (25/56, 44.6%; *p* < 0.0001) and *bla*_KPC-2_ (6/56, 10.7%; *p* < 0.0001) genes were more frequently detected in CRISPR-Cas-positive isolates with both, I-E and I-E* subtypes. The simultaneous presence of *bla*_OXA-48_ and *_bla_*_NDM-1_ genes was more frequent in CRISPR-Cas negative isolates, but this was not statistically significant. The absence of carbapenemase genes was significantly more frequent in the CRISPR-Cas negative isolates (173/340, 51.3%; *p* = 0.0296) (Table 3).

Resistance rates of antimicrobial agents were not different when CRISPR-Cas positive and CRISPR-Cas negative isolates were compared. Only the resistance rate to IPM + REL was higher in CRISPR-Cas-negative isolates (11/15, 73.3% vs. 3/7, 42.9%; *p* = 0.033) and statistically significant (Supplementary Table S1). One CR isolate, producing both NDM-1 and OXA-48 and lacking a CRISPR-Cas system, was resistant to all 18 antimicrobials tested (MICs: IPM 128 µg/mL, MEM 128 µg/mL, COL 32 µg/mL), classifying it as pan-drug resistant (PDR).

### 3.4. PFGE and MLST Analysis

**Figure 3:**
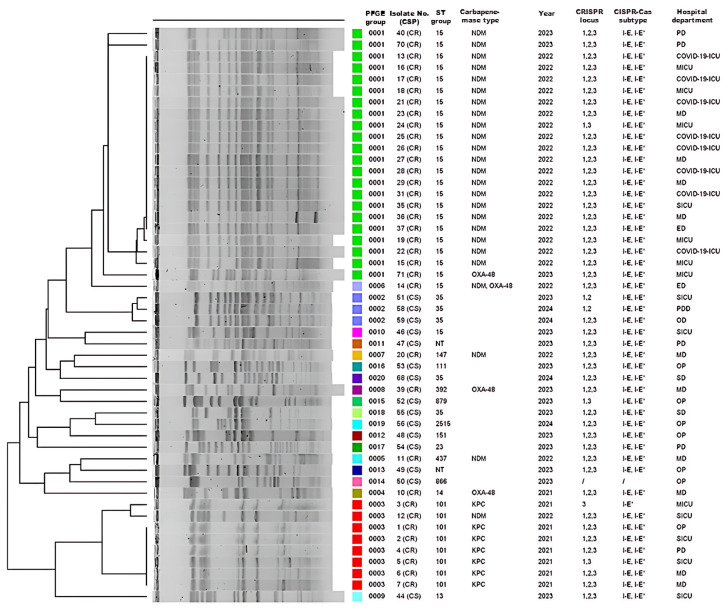
Description of 50 CRISPR-Cas-positive *Klebsiella pneumoniae* isolates according to PFGE group number, isolate number (carbapenem susceptibility profile, CSP), sampling year, sequence type (ST) groups, carbapenemase type/s, CRISPR locus types (1, 2, and/or 3), CRISPR-Cas subtype/s (I-E and/or I-E*), and hospital department. NT—non-typable isolate in which the *cas1* gene but not the CRISPR locus was detected; PD—pulmonology department; ICU—intensive care unit; MICU—medical ICU; MD—medical department; SICU—surgical ICU; ED—emergency department; PDD—pediatric department; OD—oncology department. The colored blocks in the ‘PFGE group’ column correspond to distinct pulsotypes (0001–0020), visually grouping clonally related isolates.

In order to assess genotypic diversity among 63 CRISPR-Cas-positive *K. pneumoniae* isolates, PFGE was successfully performed on 50 (15 CS and 35 CR) of the 63 CRISPR-Cas-positive isolates. These clustered into three predominant PFGE groups: cluster I comprised 22 CR isolates, cluster II three CS isolates, and cluster III eight CR isolates (Figure 3). Subsequently, MLST was conducted on a total of 39 isolates. This subset consisted of 26 representatives from the main PFGE clusters, along with the 13 isolates that could not be revived for PFGE analysis. This analysis revealed 19 distinct sequence types (STs) providing insight into the population structure of our CRISPR-positive collection.

Among the 27 CS isolates, 14 distinct STs were found, with ST35 predominating (10/27, 37.0%), followed by ST23 (3/27, 11.1%), while two isolates remained untypable. In the 36 CR isolates, we identified six STs, the most common being ST15 (23/36, 63.9%), followed by ST101 (8/36, 22.2%) and ST14 (2/36, 5.6%). PFGE and MLST demonstrated limited genetic diversity in carbapenemase-producing isolates, segregating into two PFGE clusters corresponding to *bla*_NDM-1_ (*n* = 22; ST15) and *bla*_KPC-2_ (*n* = 7; ST101) genes. A single ST group generally corresponded to a single PFGE group. An exception was the CR isolate 14, which produces both NDM-1 and OXA-48 carbapenemases and was classified as pulsotype 0013, while other CR isolates from the ST15 group, producing NDM-1, were identified as pulsotype 0012. Similarly, five isolates from the ST35 group were associated with three different macro-restriction pulsotypes (0002, 0018, and 0020), highlighting PFGE’s higher discriminatory power. PFGE analysis resolved the 50 isolates into 20 distinct pulsotypes. Subsequent MLST analysis of the 39-isolate subset identified 19 distinct STs. Three CR isolates (ST347, ST147, ST392) showed macro-restriction and ST profiles more similar to certain CS isolates than other CR isolates (Figure 3). *bla*_NDM-1_-positive ST15 isolates were largely recovered from COVID-19 departments and ICUs during 2022 (8/23, 34.8%), whereas the ST101 KPC-2 producers predominated in 2021. Among CS isolates, the ST35 group was the most prevalent in surgical departments.

### 3.5. CRISPR Polymorphism and Its Relation with Carbapenem Resistance, MLST, and Hospital Departments

From the 63 CRISPR-Cas-positive isolates, a subset of 23 strains was selected for detailed analysis of their CRISPR loci. The CRISPR1-3 loci of 23 *K. pneumoniae* isolates were sequenced, revealing varying numbers of spacers (ranging from 3 to 10), diversity, and common spacers. Each spacer is separated by a conserved direct repeat sequence of 28 base pairs (bp) (5′ GTCTTCCCCACACGCGTGGGGGTGTTTC 3′) in the CRISPR1 locus, 28 bp (5′ GAAACACCCTCACGCAGGTGGGGAAGAC 3′) in the CRISPR2 locus, and 29 bp (5′ GTCTTCCCCACGCACGTGGGGGTGTTTCT 3′) in the CRISPR3 locus [17].

CRISPR1 had a mixture of shared spacers at different positions; for example, the CRISPR1 locus of CR isolate 8 (ST14) and CS (NT) isolate 49 was identical to that of ST15 isolates. All CR isolates, however, shared an identical set of spacers in their CRISPR1 locus. CRISPR2 was highly conserved; however, the proximal spacer of CRISPR2 was shared between CR isolates (ST14, ST15) and two CS isolates (ST23 isolate 41, ST151 isolate 48), while the remaining spacers differed. In CRISPR3, the first, second or both proximal spacers were shared by several CS (41, 42, 46, 63, 68) and CR isolates (8, 10, 12, 13, 28, 70, 71) from ST23, ST35, ST306, ST14, ST15, and ST101 groups: 5′-TCCAGTACGCCAATGCTGGTAGACCCCTCACA-3′ and 5′-AGAACGAATGCCCGCGCTGGTACGGCGCGTCGTGGATTCC-3′, suggesting that the CRISPR3 locus is active and a plasmid may be spreading among the isolates to serve as a source of these spacers.

Finding a correlation between CRISPR loci composition and ST group and/or hospital ward proved difficult. For example, the CRISPR3 loci of ST35 isolates 42, 65, and 68 revealed variable spacer composition, suggesting that these strains were not clonally distributed although they originated from the same ward. On the other hand, CS isolates 56 (ST2515), 63 (ST306), and 65 (ST35) also had different spacer compositions at CRISPR3 but identical CRISPR2 spacers, suggesting that this locus may have originated from a common ancestor. Interestingly, almost all dominant ST14, ST15, and ST101 group CR isolates (8, 10, 12, 13, 15, 28, 40, 70, 71) had identical CRISPR1–3 loci, although they came from different wards. This shows that the composition of spacers within CRISPR loci of CR isolates correlates with large clonal complex STs but not with sampling location. In contrast, spacer diversity is greater in CS isolates, including sparsely distributed or non-homologous spacers, even within the same ST group. We also identified 17 non-homologous spacers (15.5%, 17/110)—four in the CRISPR1 loci of isolates 40 and 52, and thirteen in the CRISPR3 loci of isolates 12, 71, 41, 42, 56, 65, and 68, originating from four hospital departments (Table 4). These spacers are distinct from phages, plasmids, bacterial genomes, or other sequences in GenBank.

### 3.6. The Origin of CRISPR Spacers

We analyzed 110 different spacer sequences using CRISPRCasFinder and BLASTn, with a minimum threshold of 90.0% nucleotide matches. Of these, 93 spacers (84.5%) yielded homologous sequences in GenBank, with 92 (98.2%) spacers aligning with chromosomal regions of *K. pneumoniae.* Notably, 25 of these 93 spacers (26.9%) also matched sequences from other bacterial species, most frequently corresponding to *K. variicola*, *Escherichia coli*, *K. oxytoca*, *K. michiganensis*, *Raoultella ornithinolytica*, *K. aerogenes*, *K. grimontii*, *Citrobacter freundii*, and *Pseudomonas aeruginosa*. Approximately 41.9% (39/93) of spacers with significant nucleotide identities showed similarity to bacteriophages and/or plasmids (Table 5). Homologous spacer sequences have been documented worldwide, with high-throughput sequencing data predominantly reported from Norway, Australia (Melbourne), the United States, China, Russia, the United Kingdom, Spain, Germany, and Switzerland. Specifically, two spacers exhibited quadruple homology, matching *K. pneumoniae* genomes, other bacterial species, phages, and plasmids, and were found in globally distributed sequences. For instance, one such spacer from CS isolate 52 (ST879) matched over 100 GenBank sequences including plasmids, a phage, and *K. michiganensis* and *K. grimontii* isolates. Conversely, some spacers from CS isolates 48 (ST151), 52 (ST879), and 63 (ST306) were very rare globally, with one CRISPR3 spacer sequence, from isolate 63, matching only a single phage sequence (OU509537.1).

## 4. Discussion

We explored the possible association between the presence of the CRISPR-Cas systems and antimicrobial resistance of one of the most challenging bacterial pathogens, *K. pneumoniae*. In 2017, over 30,000 invasive CR *K. pneumoniae* isolates were reported in Europe. By 2019, 43.0% of countries had documented regional or interregional spread of CRE [7]. To our knowledge, this is the first study in Croatia to characterize the prevalence and dynamics of CRISPR-Cas systems in clinical *K. pneumoniae* isolates, contributing to a broader understanding of their role in the evolution and adaptation of this significant pathogen.

Our results showed that COL (22.0%), AMK (14.4%), and FDC (11.8%) exhibited the lowest resistance rates among CR isolates. Notably, CR isolates were more susceptible to colistin than to carbapenems, although optimal therapeutic strategies for this key CRE drug are still debated [3]. Amikacin showed continued therapeutic potential, while cefiderocol demonstrated the highest activity, with its novel mechanism positioning it as a strong alternative for multidrug-resistant (MDR) and extensively drug-resistant (XDR) *K. pneumoniae* strains [3]. These findings highlight antimicrobial agents’ selective activity against CR isolates and emphasize the challenges in managing infections caused by CRE [3,31]. The relationship between antimicrobial resistance and the presence of CRISPR-Cas systems is complex. While some studies in China, Iraq and Iran report an inverse correlation with antibiotic resistance [17,19,32,33], others associate these systems with hypervirulent CR *K. pneumoniae* clones linked to significantly higher mortality rates in patients in China [16] or find variable and often non-significant differences when comparing antimicrobial resistance between CRISPR-Cas positive and -negative isolates in Egypt [20]. These conflicting findings prompted us to characterize these dynamics within our local clinical *K. pneumoniae* isolate population at the UHCZ.

PCR screening of the CRISPR-Cas system signature gene *cas1* in a collection of 400 clinical isolates of *K. pneumoniae* revealed a low CRISPR-Cas prevalence of 15.8%. The most similar results to this study were described by Liao et al., where a CRISPR-Cas system prevalence of 14.9% was recorded in clinical carbapenem-resistant *K. pneumoniae* isolates in China [16]. Prevalence of CRISPR-Cas type I was also reported in Iraq (38.0%), Iran (34.2%), Taiwan (41.2%; 30.7%), Egypt (25.4%), and China (23.5%; 21.3%, and 14.9%) [16,17,18,19,20,32,34]. The limited distribution of CRISPR-Cas system observed in our study might be attributed to strong selective pressure for the acquisition of resistance or virulence genes, potentially leading to the loss of the CRISPR-Cas system, or the CRISPR-Cas system itself acting as a mobile genetic element whose distribution depends on the ST group [16,18]. The *cas1* gene was used as an indicator for the presence of the CRISPR-Cas system due to its essential role in spacer integration [18]. Interestingly, three CS isolates were found to possess the *cas1* gene without associated CRISPR loci, which might indicate locus loss through deletion, locus loss due to self-targeting spacers, or the presence of an alternative CRISPR-Cas system, such as a plasmid-borne type IV system, which is thought to lack genes encoding Cas1 and Cas2 proteins [13,14,35,36].

The CRISPR-Cas systems in this study were consistently localized to specific genomic loci. In contrast, PCR product sizes for CRISPR loci varied, demonstrating that differing base pair lengths do not inherently indicate spacer content divergence, whereas identical sizes frequently suggest spacer similarity. Subtype I-E systems carried a CRISPR1 locus, whereas subtype I-E* systems harbored CRISPR loci 2 and/or 3, which coincides with previous research [14,16,19,20]. Nearly all CRISPR-Cas-positive isolates (93.3%) contained both subtypes, which aligns with findings from China, where Hu et al. observed the coexistence of subtypes I-E and I-E* [34]. Specifically, among the CRISPR-Cas-positive isolates in our study, three CS isolates exhibited only CRISPR1 (subtype I-E), and a single CR isolate possessed only CRISPR3 (subtype I-E*). Some recent studies noted a higher prevalence of subtype I-E among *K. pneumoniae* isolates, while subtype I-E* was more common in the studies by Liao et al. and Hu et al. [16,17,20,34]. Such observations, along with the findings of our study that same spacers were identified in diverse *K. pneumoniae* isolates and other Enterobacterales, reinforcing the hypothesis that geographical variability of CRISPR-Cas systems highlights the need for monitoring, as these may influence multidrug resistance emergence [37].

Although spacer-based typing alone cannot classify *K. pneumoniae* strains as CR or CS, its combination with MLST enhanced discriminatory power. On the one hand, the evidence for this was our finding that CS isolates belonging to the same ST group (e.g., ST35) could be further differentiated by their variable CRISPR spacer compositions, thus providing a higher resolution than MLST alone. On the other hand, this study defined two CRISPR spacer-based “ST clonal complexes”: ST14, ST15, and ST101 CR strains and ST35 CS strains. CR isolates generally displayed highly conserved spacer compositions, often specific to their ST group and sampling period, suggesting clonal inheritance of these CRISPR arrays. In contrast, CS isolates, including those within the ST35 complex, exhibited considerably greater spacer diversity, even within the same ST group. Previous studies have shown similar results, with CRISPR-containing genomes mainly concentrated in ST147, ST14, ST15 MDR isolates, and especially the hypervirulent clone ST23, significantly contributing to carbapenem-resistant infections [20,34]. In agreement with previous studies, none of the STs in our study belonging to the high-risk clonal complex 258 were found to harbor the CRISPR-Cas system [19,20,34].

Our detailed analysis of 110 different spacers revealed that 84.5% of spacers showed homology to sequences in GenBank, with the majority (98.9%) matching chromosomal regions of *K. pneumoniae*. Spacers matching *K. pneumoniae* chromosomes may imply CRISPR-Cas roles beyond adaptive immunity [14]. A smaller proportion of homologous spacers matched plasmids (18.3%) and phages (29.0%), including identical spacer sequences complemented to multiple plasmids from *K. pneumoniae* isolates and other Enterobacterales. For instance, the CRISPR1 spacer 5′-CCTGCAGCTGGCCGTCGAGCTGACGGATGCCGG-3′ occurs on >24 plasmids; the CRISPR2 spacer 5′-CCGGCATCCGTCAGCTCGACGGCCAGCTGCAG-3′ on >14; and the CRISPR3 spacer 5′-TGCCGGATATCATCACCGCGATTAAACGGCGG-3′ on >94 plasmids. The CRISPR1 locus often showed shared spacers among different CR isolates, suggesting clonal spread or common selective pressures, while the CRISPR2 locus was highly conserved in specific CR strains but varied between CR and CS groups. On one hand, the highly conserved CRISPR loci within the dominant CR *K. pneumoniae* clonal complexes (ST14, ST15, ST101) suggest that these systems are potentially vertically inherited, indicating limited recent spacer acquisition. We cannot confirm whether the CRISPR3 locus of CR isolates is exclusively inherited or if it is both inherited and active, considering the identical proximal spacer shared by a significant number of CS and CR isolates. On the other hand, the greater spacer diversity found in CS *K. pneumoniae* isolates, particularly within the CRISPR3 locus, points towards a potentially functional system that is currently or was recently engaged in acquiring new spacers from MGEs. We also found that 17 CRISPR spacers showed no homology to known chromosomal, plasmid, or phage sequences. These non-homologous spacers could represent recent acquisitions from as-yet uncharacterized MGEs to which the *K. pneumoniae* isolates were exposed.

Isolates with the subtype I-E* CRISPR-Cas system in previous study demonstrated significantly greater susceptibility to ampicillin-sulbactam, cefazolin, cefuroxime, and gentamicin, possessing fewer plasmids, prophage regions, and resistance genes than CRISPR-Cas-negative isolates [19]. Conversely, our findings indicate that CRISPR-Cas-positive isolates non-typically showed non-significantly higher levels of antibiotic resistance for all three carbapenems, except for the resistance rate of IPM + REL that was significantly higher in CRISPR-Cas-negative isolates (*p* = 0.033). This atypical trend could suggest that these CR isolates represent specific clonal lineages, with I-E and I-E* subtypes. Similar observations have been made in studies identifying hypervirulent clones with particular CRISPR-Cas subtype I-E* [19]. More specifically, patients with CRISPR-Cas-positive CR *K. pneumoniae* isolates also showed a higher incidence of comorbidities like hypertension and diabetes. This is likely due to many CRISPR-Cas systems being carried by clonally disseminating CR isolates.

For instance, CRISPR-Cas-positive ST15 NDM-1-producing strains in our study demonstrated probable monoclonal spread especially among adult patients in COVID-19 departments and ICUs. A similar pattern was observed with ST101 CR strains that produced KPC-2, but these strains originated from different departments. In contrast, CRISPR-Cas-negative isolates are more common in pediatric ICU patients and stool samples. This variation suggests that the CRISPR-Cas system may influence bacterial resistance and relate to specific clinical settings and patient populations. Adult patients may represent a more appropriate target population for CRISPR-Cas system testing due to their greater exposure to foreign genetic elements. The presence of identical spacers in the CRISPR1-3 loci of predominantly CR isolates may indicate exposure to the same foreign genetic material or clonal inheritance of the CRISPR-Cas system instead of active functionality. This aligns with findings in *Salmonella* and *E. coli*, where CRISPR-Cas systems were showing limited capacity to prevent plasmid transfer and the spread of antibiotic resistance [14].

Our molecular analyses indicate that among CR isolates with carbapenemase genes, the *bla*_OXA-48_ gene was significantly more prevalent in CRISPR-Cas-negative strains. The *bla*_OXA-48_ gene is exclusively associated with conjugative plasmids IncL/M (~60 kb), which rarely contain additional resistance genes [38], suggesting a negative impact of CRISPR-Cas type I on the acquisition of these plasmids. Research in China also revealed that the dominant *bla*_KPC-2_ carbapenemase gene was the most prevalent in CRISPR-Cas-negative strains compared to those with subtype I-E or I-E* [16]. In contrast, *bla*_NDM-1_ and *bla*_KPC-2_ genes in our study were significantly (*p* < 0.0001) more frequently detected in CRISPR-Cas-positive isolates with both I-E and I-E* subtypes. This observation is supported by Kadkhoda et al., reporting a significantly higher frequency of the *bla*_NDM-1_ gene in isolates harboring the subtype I-E [39]. Perhaps *bla*_KPC-2_-bearing IncFII plasmids can coexist with type I-E* CRISPR-Cas systems in ST15 *K. pneumoniae*, as proposed by Hu et al. [34]. This differential association was evident in our CR isolates belonging to ST14, ST15, and ST101, which, despite having homogeneous CRISPR loci profiles, seemed to effectively prevent the acquisition of *bla*_OXA-48_-carrying plasmids but not those carrying *bla*_NDM-1_ or *bla*_KPC-2_. An interesting case was a CS isolate 46 (ST15), which, despite being carbapenem susceptible, shared a CRISPR profile with CR ST15 isolates. This could indicate an active CRISPR-Cas system against other mobile genetic elements or a prior elimination of resistance plasmids.

Therefore, we concluded that the coexistence of CRISPR-Cas systems and numerous antibiotic resistance genes (ARGs) in *K. pneumoniae* is more complex than a simple inverse relationship. In addition, Alkompoz et al. found that specific ARGs, such as *bla*_VIM_ and *bla*_NDM_, are more prevalent in CRISPR-Cas-positive strains, whereas others (e.g., *bla*_KPC_) are more common in CRISPR-Cas-negative genomes [20]. These findings imply that CRISPR-Cas-mediated suppression of carbapenemase genes likely depends on CRISPR loci composition and plasmid features, where incomplete arrays or specific PAM sequences may disrupt adaptive interference [25]. Indeed, the functionality of CRISPR-Cas systems can be modulated by various factors, including anti-CRISPR (Acr) proteins encoded by phages or plasmids; mutations in *cas* genes or PAM sequences can impair CRISPR function and facilitate multidrug resistance; the system’s efficacy is influenced by spacer GC content and its proximity to the leader sequence, which is critical for transcriptional regulation; host regulatory mechanisms, such as the H-NS protein activated by high concentrations of imipenem, can bind the CRISPR-Cas operon promoter, inhibiting its activity and suppressing *cas3* expression; and other defense immune mechanisms, including restriction-modification (R-M) systems [14,16,18,20,25,40]. These findings underscore the multifaceted nature of CRISPR-Cas interactions with ARGs, highlighting that various genetic and environmental factors determine its impact on antibiotic resistance spread.

Our study has two limitations. Firstly, MLST was performed only on selected isolates, and secondly, whole-genome sequencing would be valuable addition to this CRISPR-Cas investigation.

## 5. Conclusions

The absence of CRISPR-Cas systems in clinical *K. pneumoniae* isolates with *bla*_OXA-48_ suggests a lack of protection in these isolates against *bla*_OXA-48_ acquisition. Conversely, *bla*_NDM-1_ and *bla*_KPC-2_ were more frequently detected in CRISPR-Cas-positive isolates. Our findings highlight a potential dual dynamic of CRISPR-Cas evolution in *K. pneumoniae* isolates. While the stability of CRISPR arrays in dominant CR *K. pneumoniae* clonal complexes like ST15 and ST101 potentially points to clonal inheritance, the significant spacer diversity found in CS *K. pneumoniae* isolates suggests that the CRISPR-Cas system probably remains active in other genetic backgrounds. The compatibility between CRISPR loci composition and dominant clonal complexes like ST14, ST15, and ST101 in CR isolates suggests that CRISPR loci could be valuable tools for subtyping following MLST. However, further research is needed to better understand the relationship between CRISPR-Cas systems, antibiotic resistance, and sequence type distribution. Whole-genome sequencing of a more extensive and diverse collection of carbapenem-susceptible and -resistant isolates, including both CRISPR-Cas-positive and -negative strains, is essential to fully elucidate their role in resistance mechanisms and clonal dissemination. Furthermore, exploring the prevalence of anti-CRISPR proteins in local *K. pneumoniae* isolates could help explain instances where CRISPR-Cas systems fail to prevent the acquisition of new plasmids.

## Figures and Tables

**Table 1:** Distribution of collected clinical samples according to the hospital departments (*n* = 400).

Department	Number of Samples (%)
Medical Departments	64 (16.0)
Medical Intensive Care Units	57 (14.3)
Pediatric Intensive Care Units	51 (12.8)
Surgical Intensive Care Units	45 (11.3)
Surgery Departments	44 (11.0)
Outpatients	40 (10.0)
Hematology Departments	25 (6.3)
Pulmonology Departments	23 (5.8)
Emergency Departments	21 (5.3)
COVID-19 Intensive Care Units	12 (3.0)
Pediatric Departments	9 (2.3)
Oncology Departments	6 (1.5)
COVID-19 Departments	3 (0.8)

**Table 2:** The sequences of primers used in this study.

Gene/CRISPR Locus	Primer Sequence (5′-3′)	Annealing Temperature (°C)	PCR Product Size (bp)
*cas1*	F-GCTGTTTGTCAAAGTTACCCGCGAACTC	58	150
	R-GGTTTTGATCGCCTCATGAGTCACAGTTG		
*rpoB*	F-GGCGAAATGGCWGAGAACCA	54	1100
	R-GAGTCTTCGAAGTTGTAACC		
I-E CRISPR1	F-GACGGTGGTTATATGGTGAC	52	Variable (400–900)
	R-CATTGATGCCTCTACGTCAG		
I-E* CRISPR2	F-GTAGCGAAACCCTGATCAAGCG	57	Variable (900–1300)
	R-GCGCTACGTTCTGGGGATG		
I-E* CRISPR3	F-GACGCTGGTGCGATTCTTGAG R-CGCAGTATTCCTCAACCGCCT	58	Variable (1300–2100)

W—stands for adenine or thymine in degenerative primers (*Weak*); bp—base pair.

**Table 3:** Comparison of occurrence of carbapenemase genes and presence of the CRISPR-Cas system in 400 *Klebsiella pneumoniae* bacterial isolates.

Carbapenemase Gene	CRISPR-Cas System					*p*-Value
	*cas1* Without Loci (*n* = 3)	Type I-E (*n* = 3)	Type I-E* (*n* = 1)	Types I-E and I-E* (*n* = 56)	Absent (*n* = 337)	
*bla*_NDM_ (*n* = 53)	0	0	0	25 (44.6%)	28 (8.3%)	<0.0001
*bla*_KPC_ (*n* = 10)	0	0	1 (100%)	6 (10.7%)	3 (0.9%)	<0.0001
*bla*_OXA-48_ (*n* = 123)	0	0	0	3 (5.3%)	120 (35.6%)	<0.0001
*bla*_OXA-48_ and *bla*_NDM_ (*n* = 14)	0	0	0	1 (1.8%)	13 (3.9%)	0.9290
without carbapenemase gene (*n* = 200)	3 (100%)	3 (100%)	0	21 (37.5%)	173 (51.3%)	0.0296

**Table 4:** Sequences of CRISPR spacers with no homology with matching plasmids, phages, and chromosomes regarding the CRISPR locus, carbapenem susceptibility profile (CSP), ST group, and hospital department.

CRISPR Locus	Spacer Sequence (5′ to 3′)	Isolate No. (CSP)	ST Group	Hospital Department
CRISPR3	GCAGTCGATTCGTCTCGACAGTGACAGGGGC	12 (CR)	ST101	Surgical intensive care units
CRISPR1	TCTTGCTTGGAGAGGATTCTACAGTCTCACCAT	40 (CR)	ST15	Pulmonology departments
CRISPR1	TACGTCCCGAAGACCACTGCCGGGGGATCAAGG			
CRISPR1	CTGGGGGCAGGACCCCGATATGACGGAGGTGA			
CRISPR3	TGTGTGACAAAGCCACGTCCGGGAAGAACAAT	41 (CS)	ST23	Surgery departments
CRISPR3	AAACTGCGCATCGTTCGACGCGAGCGACATCGA	42 (CS)	ST35	Outpatients
CRISPR1	CGTGTTTTTGTGTTTAATGGTCATTTATGATTT	52 (SC)	ST879	Outpatients
CRISPR3	ACACGAAATGACGGGGTTTCGCCGGTCGTCTCA	56 (CS)	ST2515	Outpatients
CRISPR3	CCGGCTACAATGCGATCGGTGGGCAGTGGTTGC			
CRISPR3	AAAACCCAGTAGACGGGGATAGAGACAAAAG	65 (CS)	ST35	Surgery departments
CRISPR3	TACGTGGAATACCGTGTTGCACCAATGAATATG			
CRISPR3	AAATTCAGCAGGTCGCGGGATGCCGTGGTTGT	68 (CS)	ST35	Surgery departments
CRISPR3	ACCGCGATCCGTTCCGGCTTAGGCCGTTTAT			
CRISPR3	CCGTCATTCATATTTCCGGGGAAACTGGGTT			
CRISPR3	CAACGAAGTAAACGGGGATCGTCCGTCCAAGA			
CRISPR3	GGCACCCCTTCCGCCCAAAGGGGCCCTATTCTA	71(CR)	ST15	Medical intensive care units
CRISPR3	AAACCAATCAAAATTCTTCCCCACATGGAAAGTGGCCA			

**Table 5:** CRISPR-Cas spacers sequences matching plasmids and/or phages sequences.

Spacer Sequence (5′ to 3′)	Isolate No.	Phage (Accession No.)	Plasmid (Accession No.)	Homology Type
CRISPR1 Locus				
TGCCTCCAATGCAATCACCGGCCTGCTAACCGG	8, 40, 49, 71, 46, 28	BK047705.1	/	Phage
CGTCATCAGCGCCTTGTTCCAGCGGCGACCACC	8, 40, 49, 54, 71, 46, 28	NC_071011.1	/	Phage
CCTGCAGCTGGCCGTCGAGCTGACGGATGCCGG	8, 49, 53, 54, 71, 46, 28	/	AP024174.1	Plasmid
TGTAGCGCGGCTGGTTGATGCACTGAGGCACTA	52	MK422452.1	/	Phage
CAAATGGGAGAAGCTAATCGTTGGGGTGCTGAA	52	MK416022.1	OW848780.1	Phage and Plasmid
TGGTCATCGCGCCCCTTGGCCTGCTCTGCGCTG	53	MN013086.1	/	Phage
TCCTTCATTAAGTGAGCAATTGCTTCCTTTTTT	53	PP934564.1	/	Phage
CGGCTCTTTTTTATCTCCTTCATCCTTCGCTAT	54	BK055976.1	/	Phage
TGATCGGCGTGCCGTTTGTTGGACCCGAAATAG	54	BK031684.1	/	Phage
CGAGCTCATCGCCTCCCTGGAGACGGCGGGCGA	63, 65	BK031364.1	/	Phage
CAAGACACCTGCAAACGGTATATCTTTGGAGTG	63, 65	OP617741.1	/	Phage
**CRISPR2 Locus**				
AGGATAGAGCCAAATCCGCTCACACGTGATGA	15, 48, 8, 12, 71, 41, 28, 46	/	CP079158.1	Plasmid
CCGGCATCCGTCAGCTCGACGGCCAGCTGCAG	15, 48, 8, 12, 71, 41, 28, 46	/	CP081815.1	Plasmid
ACGTGATCGCCCTGGCGCGGACGCCGGGAGGT	15, 48, 8, 12, 71, 41, 28, 46	NC_071011.1	CP079158.1	Phage and Plasmid
ATGGTGCGACTGTAGAATCCTCACCATGCACG	15, 48, 8, 12, 71, 41, 28, 46	NC_071011.1	CP079158.1	Phage and Plasmid
GATAATCCCGTCAGGTTGTGACTCTGCACGAT	15, 48, 8, 12, 71, 41, 28, 46	NC_071011.1	CP079158.1	Phage and Plasmid
TCGAGGACATTACCGAGGACTACGACGACTGG	15, 48, 8, 12, 71, 41, 28, 46	BK019580.1	/	Phage
GGTGGTCGCCGCTGGAACAAGGCGCTGATGAC	15, 48, 8, 12, 71, 41, 28, 46	NC_071011.1	CP079158.1	Phage and Plasmid
GACTGGCTCGGCTACGAGGTGCGCTTCGACAC	15, 8, 12, 71, 28, 46	/	CP079158.1	Plasmid
CCGGTTAGCAGGCCGGTGATTGCATTGGAGGC	15, 8, 12, 71, 28, 46	BK047705.1	CP079158.1	Phage and Plasmid
CTATTTCGGGTCCAACAAACGGCACGCCGATC	41	BK031684.1	CP093490.1	Phage and Plasmid
ATAGCGAAGGATGAAGGAGATAAAAAAGAGCC	41	BK055976.1	/	Phage
CCACTCCAAAGATATACCGTTTGCAGGTGTCTTG	56, 65, 63	OP617741.1	/	Phage
TCGCCCGCCGTCTCCAGGGAGGCGATGAGCTCG	56, 65, 63	BK031364.1	/	Phage
**CRISPR3 Locus**				
TCCAGTACGCCAATGCTGGTAGACCCCTCACA	8, 10, 12, 13, 28, 41, 42, 46, 63, 68, 70, 71	/	CP183011.1	Plasmid
AGAACGAATGCCCGCGCTGGTACGGCGCGTCGTGGATTCC	8, 10, 12, 13, 28, 46, 63, 70, 71	/	CP079158.1	Plasmid
ATTCAGCTGAAAACTGCCAGTATCGCGGCGGT	8, 10	/	CP079675.1	Plasmid
TCGCCGTCGAAGTGCTGCGCGATAGGGATGAT	8, 10	/	CP079675.1	Plasmid
TGCCGGATATCATCACCGCGATTAAACGGCGG	12, 70, 13, 28, 46	/	CP067552.1	Plasmid
GCTAACCAGTGGATAGAGCACTATGTGACGAC	12, 70, 13, 28, 46, 71	BK049466.1	/	Phage
GCTACTGCATCCACGGCGTACATGCTCAGTGT	12, 70, 13, 28, 46, 71	BK052889.1	/	Phage
CTTCGACACCAACCCAAACAGATCTGGCCTGGA	41	BK049466.1	/	Phage
TGGAATTTTCGCGTCTCCAAAAACTGCGCATC	42	BK052641.1	/	Phage
GCGGACGCGCTCCATGAAGTAGTCCCGCAGGT	42	BK056588.1	/	Phage
CCGCAGCCGCGGCGGCTTTTGCCGGTGCTGAC	63	MN013086.1	/	Phage
TGCCTGCTGATCTGCGGCGTTATCTGGACAGCAG	63	OR532892.1	/	Phage
TGTCCCGGATACGCTTTCCGCCATTGATGCGC	63	OU509537.1	/	Phage
GCTAACCAGTGGATAGAGCACTATGTGACGAC	71	BK049466.1	/	Phage
GCTACTGCATCCACGGCGTACATGCTCAGTGT	71	BK049466.1	/	Phage

The ‘Homology Type’ column is color-coded to visually represent the type of genetic element matched by the spacer sequences: green indicates homology to phages, orange indicates homology to plasmids, and red indicates homology to both bacteriophages and plasmids.

## Data Availability

Data are not publicly available due to privacy and ethical restrictions. Patient data will not be shared with third parties. Further inquiries can be directed to the corresponding author.

## Supplementary Materials

The following supporting information can be downloaded at: https://www.mdpi.com/article/10.3390/pathogens14060604/s1, Table S1: Comparison of antimicrobial resistance rate between CRISPR-Cas-positive and CRISPR-Cas-negative *Klebsiella pneumoniae* isolates.

## Abbreviations

The following abbreviations are used in this manuscript:

AMC	Amoxicillin + clavulanic acid
AME	Aminoglycoside-modifying enzymes
AMK	Amikacin
AMP	Ampicillin
AMR	Antimicrobial resistance
AMX	Amoxicillin
BLAST	Basic Local Alignment Search Tool
bp	Base pairs
CAZ	Ceftazidime
CAZ + AVI	Ceftazidime + avibactam
CFM	Cefixime
CIP	Ciprofloxacin
CLX	Cephalexin
COL	Colistin
CPD	Cefpodoxime
CR	Carbapenem resistant
CRE	Carbapenem-resistant Enterobacterales
CRO	Ceftriaxone
CS	Carbapenem susceptible
CSP	Carbapenem susceptibility profile
CTX	Cefotaxime
CXM	Cefuroxime
ED	Emergency department
ESBL	Extended-spectrum β-lactamase
ETP	Ertapenem
EUCAST	European Committee on Antimicrobial Susceptibility Testing
FDC	Cefiderocol
FEP	Cefepime
GEN	Gentamicin
HGT	Horizontal gene transfer
ICU	Intensive care unit
IPM	Imipenem
IPM + REL	Imipenem + relebactam
LVX	Levofloxacin
MD	Medical department
MEM	Meropenem
MGE	Mobile genetic element
MIC	Minimum inhibitory concentration
MICU	Medical intensive care units
MLST	Multilocus sequence typing
n	Number
N/A	Not applicable
NT	Non-typable
OD	Oncology department
OP	Outpatients
PCR	Polymerase chain reaction
PD	Pulmonology departments
PFGE	Pulsed-field gel electrophoresis
PDD	Pediatric department
SD	Surgery departments
SICU	Surgical intensive care units
ST	Sequence type
SXT	Trimethoprim + sulfamethoxazole
TOL + TAZ	Ceftolozane + tazobactam
TZP	Piperacillin + tazobactab
W	Weak
