# Supplementary material for: CRISPR-Cas Dynamics in Carbapenem-Resistant and Carbapenem-Susceptible Klebsiella pneumoniae Clinical Isolates from a Croatian Tertiary Hospital
Source: Pathogens. 2025 Jun 19;14(6):604. doi: 10.3390/pathogens14060604 (PMC12195874; doi:10.3390/pathogens14060604)
Supplement: Supplementary file 1 [file pathogens-14-00604-s001.zip › pathogens-3701107-supplementary.pdf]

Supplementary Table S1. Comparison of antimicrobial resistance rate between CRISPR-Cas-positive and CRISPR-Cas-negative *Klebsiella pneumoniae* isolates.

**Antimicrobial agent (*n*)**	**CRISPR-Cas-negative *K. pneumoniae* isolates (*n* = 337)**		**CRISPR-Cas-positive *K. pneumoniae* isolates (*n* = 63)**		**p-value**
	**Number of tested isolates**	**Resistance rate (%)**	**Number of tested isolates**	**Resistance rate (%)**	
ampicillin/amoxicillin (*n* =400)	337	100.0	63	100.0	N/A
amoxicillin+clavulanic acid (*n* =400)	337	49.0	63	57.1	N/A
cephalexin (*n* =143)	122	49.2	21	42.9	0.6632
cefuroxime (*n* =399)	336	48.2	63	57.1	1.0000
ciprofloxacin (*n* =400)	337	48.7	63	60.3	N/A
levofloxacin (*n* =397)	335	46.3	62	59.7	0.4028
cefpodoxime (*n* =148)	127	50.4	21	42.9	0.5119
cefixime (*n* =146)	125	48.8	21	42.9	0.5700
gentamicin (*n* =400)	337	29.7	63	42.9	N/A
amikacin (*n* =376)	318	14.5	58	13.8	0.5603
trimethoprim+sulfamethoxazole (*n* =312)	263	44.1	49	59.2	0.9630
ceftazidime (*n* =373)	315	49.8	58	60.3	0.5945
cefotaxime (*n* =373)	315	50.5	58	62.1	0.5945
ceftriaxone (*n* =372)	314	50.3	58	60.3	0.7873
cefepime (*n* =374)	316	50.3	58	60.3	0.5810
piperacillin+tazobactam (*n* =373)	315	51.4	58	63.8	0.5945
ertapenem (*n* =400)	337	48.7	63	57.1	N/A
imipenem (*n* =373)	316	22.5	57	47.4	0.3396
meropenem (*n* =373)	316	34.8	57	49.1	0.3396
colistin (*n* =200)	164	14.6	36	55.6	0.2173
ceftazidime+avibactam (*n* =185)	155	20.0	30	50.0	0.8125
ceftolozane+tazobactam (*n* =109)	96	90.6	13	92.3	0.1994
imipenem+relebactam (*n* =22)	15	73.3	7	42.9	0.0335
cefiderocol (*n* =17)	16	12.5	1	0.0	0.4920

N/A – p-value is not applicable when all isolates (*n* = 400) were tested for all antimicrobial agent (*n* = 400)
